# A standardized technique for high-pressure cooling of protein crystals

**DOI:** 10.1107/S2059798317016357

**Published:** 2017-11-22

**Authors:** David Quirnheim Pais, Barbara Rathmann, Juergen Koepke, Cveta Tomova, Paul Wurzinger, Yvonne Thielmann

**Affiliations:** a Molecular Membrane Biology, Max Planck Institute of Biophysics, Max-von-Laue-Strasse 3, 60438 Frankfurt am Main, Germany; b Leica Microsystems Vienna, Hernalser Hauptstrasse 219, 1170 Vienna, Austria

**Keywords:** high-pressure cooling, protein crystals, polyimide capillary, buffer composition

## Abstract

Cryogenic temperatures slow down secondary radiation damage during data collection from macromolecular crystals. In 1973, cooling at high pressure was identified as a method for cryopreserving crystals in their mother liquor [Thomanek *et al.* (1973 ▸). *Acta Cryst.* A**29**, 263–265]. Results from different groups studying different crystal systems indicated that the approach had merit, although difficulties in making the process work have limited its widespread use. Therefore, a simplified and reliable technique has been developed termed high-pressure cooling (HPC). An essential requirement for HPC is to protect crystals in capillaries. These capillaries form part of new sample holders with SPINE standard dimensions. Crystals are harvested with the capillary, cooled at high pressure (220 MPa) and stored in a cryovial. This system also allows the usage of the standard automation at the synchrotron. Crystals of hen egg-white lysozyme and concanavalin A have been successfully cryopreserved and yielded data sets to resolutions of 1.45 and 1.35 Å, respectively. Extensive work has been performed to define the useful working range of HPC in capillaries with 250 µm inner diameter. Three different 96-well crystallization screens that are most frequently used in our crystallization facility were chosen to study the formation of amorphous ice in this cooling setup. More than 89% of the screening solutions were directly suitable for HPC. This achievement represents a drastic improvement for crystals that suffered from cryoprotection or were not previously eligible for cryoprotection.

## Abstract 2

A standardized technique for high-pressure cooling of protein crystals has been developed that encompasses all steps from crystal retrieval to automated mounting of the sample at the synchrotron. A wide chemical space for sample cooling has been tested. Amorphous ice was formed in more than 89% of the solutions.

## 1. Introduction   

Cooling of macromolecular crystals is a standard procedure in X-ray crystallography. The low temperature of 100 K slows down the process of radiation damage, which is especially useful for synchrotron radiation (Ravelli & Garman, 2006 ▸). All of the crystallization screening conditions already contain chemicals that act as cryoprotectants; often the addition of another cryoprotecting agent is necessary to form amorphous ice at cryogenic temperatures. Today, various methods for the cryoprotection of macromolecular crystals exist. The most common procedure is the use of small additives such as glycerol, ethylene glycol or 2-methyl-2,4-pentanediol (Garman & Mitchell, 1996 ▸; Vera *et al.*, 2011 ▸; Farley *et al.*, 2014 ▸; Pflugrath, 2015 ▸). Sensitive crystals often do not tolerate the high concentrations of small additives which are necessary for cryoprotection. Therefore, different approaches have used sugars (Haas & Rossmann, 1970 ▸), salts (Holyoak *et al.*, 2003 ▸; Rubinson *et al.*, 2000 ▸), low-molecular-weight PEGs (Discipio *et al.*, 1998 ▸), oils (Kwong & Liu, 1999 ▸) and mixtures (Vera & Stura, 2014 ▸). The procedure of finding a cryoprotectant solution can be very laborious and time-consuming. The changed chemical environment may influence the quality of the crystal and thus its diffraction.

A different alternative for the cryoprotection of macromolecular crystals is cooling at high pressure. The main advantage of cooling at pressures of 220 MPa is the formation of high-density amorphous (HDA) ice. Cooling causes the unit cell of the protein to shrink by 2–7%, probably owing to rearrangement in the lattice. The protein molecule itself contracts as well by 1–2%, but contracts less compared with the unit cell (Juers & Matthews, 2001 ▸). This observation means that the volume reductions for water to amorphous ice and for the unit cell of the protein are similar. In contrast, cooling of the crystal (at ambient pressure) creates low-density amorphous (LDA) ice. The phase transition from water to LDA ice increases the volume by 6.7% (Kim *et al.*, 2005 ▸). If this tendency to volume increase cannot be alleviated by the addition of a cryoprotective agent, the crystal is disrupted or has increased mosaicity. However, the cryoprotective agent may itself dissolve or compromise the order of the crystal during the cryosoak.

The method of cooling at high pressure for protein crystallography was first introduced by Thomanek and coworkers (Thomanek *et al.*, 1973 ▸). They were able to high-pressure cool myoglobin crystals at 250 MPa and 77 K in isopentane and liquid nitrogen. Since a great effort was needed for each crystal, the method was not widely pursued in crystallography. In 1984 Mishima and coworkers found that high-density amorphous ice can be created by pressures of 1000 MPa at 77 K (ρ = 1.17 g cm^−3^; Mishima *et al.*, 1984 ▸). The method of cooling at high pressure for protein crystals was rediscovered in 2005 by Kim and coworkers using helium gas for pressurization and cooling again with liquid nitrogen (Kim *et al.*, 2005 ▸). The method required 35 min for pressurization, cooling and pressure release. Later, it was further extended to include derivatization with noble gases for phasing (Kim *et al.*, 2006 ▸). The time per cooling cycle was optimized by van der Linden *et al.* (2014 ▸); here, a pressurization time of 5 min with helium gas was needed.

In the field of electron microscopy (EM), cooling at high pressure has been pursued since the 1970s after the developments of Riehle & Hoechli (1973 ▸). Continuous improvements in method development have led to technically mature high-pressure cooling devices, and these have been used over the years (Baumeister, 1982 ▸; Hohenberg *et al.*, 1994 ▸; Studer *et al.*, 1995 ▸). These developments have also been employed in the field of crystallography. Various crystal structures of crystals cooled at high pressure have been determined, ranging from small soluble proteins to large membrane proteins and a virus crystal (*Gallus gallus* lysozyme, *Canavalia ensiformis* concanavalin A, *Trichoderma longibrachiatum* xylanase, *Sus scrofa* insulin, *Escherichia coli* AcrB, *Thermosynecho­coccus elongatus* photosystem II and *Bos taurus* entero­virus 2; Burkhardt *et al.*, 2012 ▸, 2013 ▸; Kurz *et al.*, 2012 ▸). Once the crystal had been inserted into various kinds of capillaries, cooling at high pressure was feasible but tricky. A great effort was required to keep the crystals cool and to transfer them safely onto the goniometer at the synchrotron. Each quartz capillary with a high-pressure cooled crystal needed to be glued individually to a nylon loop at 135 K (Burkhardt *et al.*, 2012 ▸). This method was applied to different targets as a proof of concept, but could not be used to easily cryoprotect a large number of different crystals.

**Figure 1:**
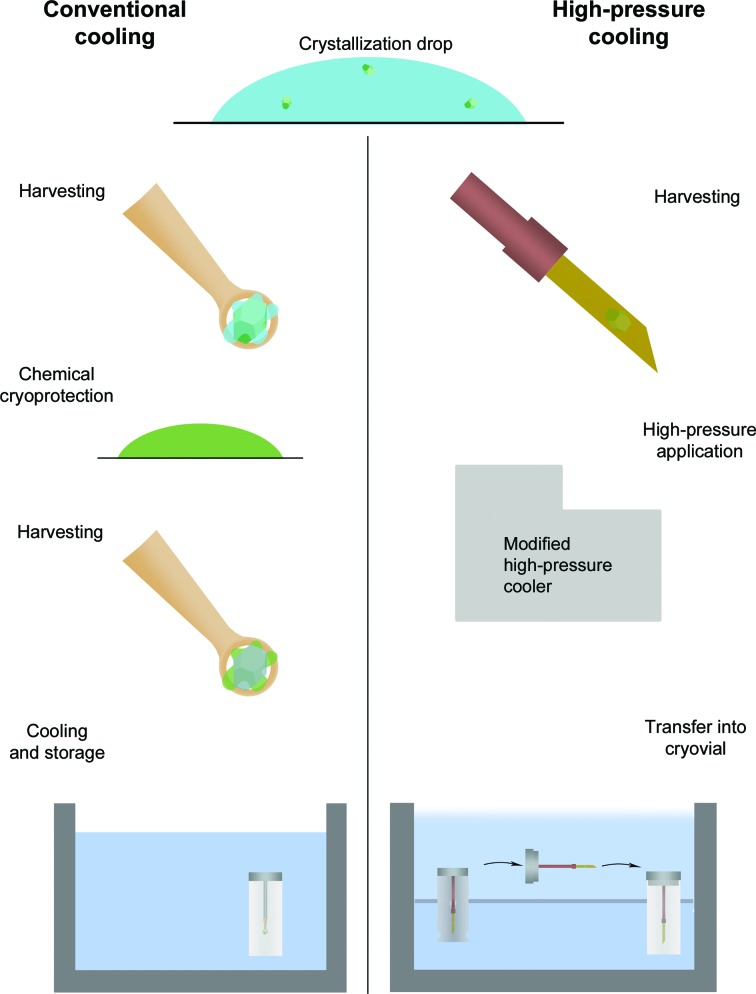
Comparison of the conventional cooling and HPC workflows. For conventional cooling the crystals are harvested in a loop. The crystal is submerged into a chemical cryocondition and harvested again. Within a few seconds the crystal is placed into a cryovial filled with liquid N_2_ or placed into a cryostream. For HPC the crystal is harvested once with the capillary of the sample unit. It is now protected by its own mother liquor. Cooling is started and the sample can be taken from the sample dewar to the semi-dry liquid N_2_ bath. The complete sample holder is formed, which is stored in a cryovial.

The cryoprotection of macromolecular crystals is not an easy task. The requirements for cooling and cryoprotection of different crystals are as variable as the crystallization process itself. Therefore, an additional reliable and rapid method is required to cryoprotect macromolecular crystals where the cooling with supplemented cryoprotectants is difficult and time-consuming or is not possible at all. To overcome these drawbacks, a standardized technique for HPC of crystals has been developed. The workflow of the method in comparison to cooling with the addition of cryoprotectants is illustrated in Fig. 1 ▸. Crystals are harvested in a capillary, inserted into the high-pressure machine and cooled. The samples drop into a dewar containing liquid N_2_. Finally, the pin and capillary are placed in a cryovial for storage and transport. At the synchrotron the high-pressure-cooled crystals can be mounted automatically just as in the regular SPINE (https://www.embl.fr/spinesampleholder/) sample holders.

## 2. Materials and methods   

### 2.1. Proteins and crystallization   

Hen egg-white lysozyme was purchased from Hampton Research (Aliso Viejo, California, USA). Concanavalin A (type IV) was purchased from Sigma–Aldrich (Steinheim, Germany). Lysozyme was dissolved in 0.02 *M* sodium acetate pH 4.6 to yield a concentration of 20 mg ml^−1^, and concanavalin A was dissolved in 0.025 *M* HEPES pH 7.0 at a concentration of 10 mg ml^−1^. They were crystallized by the sitting-drop method at 291 K in 200 nl drops at a 1:1 mixing ratio in the MPI Tray (Rathmann *et al.*, 2017 ▸). For lysozyme the reservoir solution consisted of 0.8 *M* sodium chloride, 0.1 *M* sodium acetate pH 4.6. Concanavalin A was crystallized using 6%(*w*/*v*) PEG 8000, 0.1 *M* Tris pH 8.5 as the reservoir solution.

### 2.2. Crystal harvesting   

**Figure 2:**
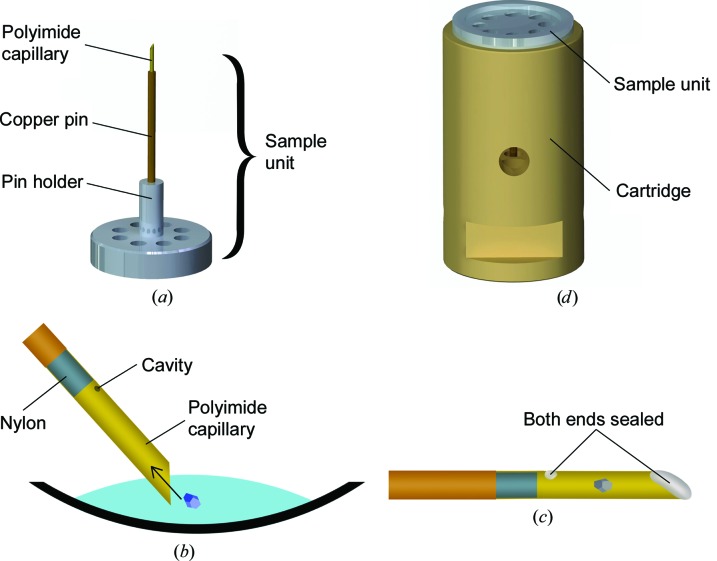
Overview of the parts and actions required before high-pressure cooling. (*a*) The sample unit consists of a polyimide capillary, a copper pin and a pin holder. (*b*) The crystal is harvested from the mother liquor with the capillary. (*c*) The capillary is filled with solution and crystal and sealed with saturated PEG 100 000 solution. (*d*) The sample unit with solution and crystal is inserted into the cartridge. This takes place in the loading station of the high-pressure cooler and is guided by a loading groove and a cylindrical tool.

To fit the needs of the high-pressure cooler, crystals were harvested with specially fabricated sample units (Fig. 2 ▸ *a*). Instead of handling crystals with loops or meshes, they were absorbed using capillary forces. A polyimide capillary with 250 µm inner diameter was purchased from Goodfellow GmbH (Bad Nauheim, Germany). The capillary was cut to form an angled tip and to a length of 5 mm. A nylon thread (Berkley, Columbia, USA) with a diameter of 220 µm was inserted into the polyimide capillary. The capillary was attached to a copper pin. The nylon thread protrudes about 1 mm from the copper pin and within the polyimide capillary. This is the limit to which the crystals can be absorbed. A cavity was pierced with a Minutien pin (http://www.fiebig-lehrmittel.de; 0.1 mm diameter) into the polyimide capillary at the end of the nylon thread (Fig. 2 ▸ *b*). The copper pin itself was mounted on a pin holder. This overall assembly is termed the sample unit. It can be extended after the cooling process to fit the SPINE standard for automated sample mounting (the sample unit together with the extension is called the sample holder). The copper pin, the pin holder and the extension were provided by Leica Microsystems Vienna (Vienna, Austria). A schematic showing the dimensions of the sample unit can be found in Supplementary Fig. S1. The sample units were pre-treated with 80%(*v*/*v*) ethanol in an ultrasonic bath and dried on a heat plate. This changed the wettability of the polyimide capillary remarkably. To harvest a crystal, the capillary was inserted into the mother liquor, pointing towards a crystal. As soon as the opening of the capillary had been submerged into the drop completely, the crystal was absorbed together with its mother liquor as shown in Fig. 2 ▸ 1. To protect the solution and crystal in the capillary, both ends of the capillary were closed immediately before cooling. A drop of a saturated solution of PEG 100 000 (the solution was extremely viscous and amenable for cleaning) was deposited onto the angled tip and cavity using an insect needle (http://www.fiebig-lehrmittel.de; 0.25 mm diameter). This could be applied very precisely and worked in more than 95% of the samples. Used sample units were cleaned with 2%(*v*/*v*) Decon 90 (Decon Laboratories Ltd, England) in an ultrasonic bath, rinsed with deionized water, treated again with ethanol and dried.

### 2.3. High-pressure cooling   

**Figure 3:**
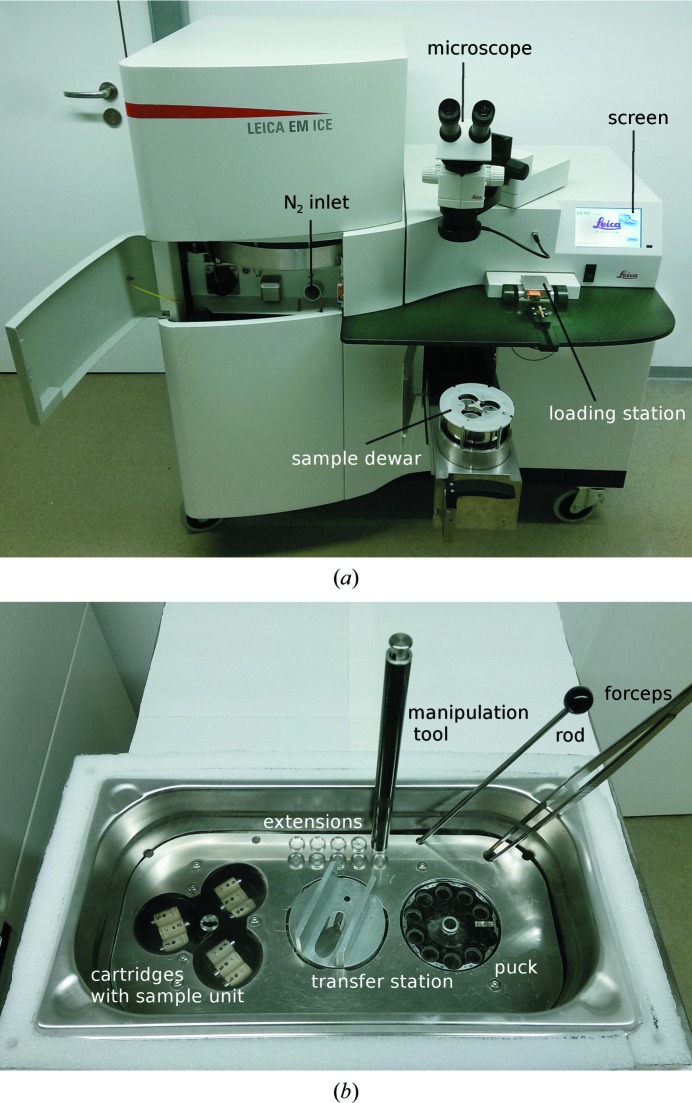
Modified high-pressure cooler and semi-dry liquid N_2_ bath. A modified Leica EM ICE is used to high-pressure cool the crystals in capillaries (*a*). From left to right: the liquid N_2_ inlet, a microscope, the sample dewar that is pulled out of the machine, a loading groove with the loading station within the table and a screen to monitor the process. Once the samples have been vitrified and stored in the sample dewar they need to be transferred to the semi-dry liquid N_2_ bath (*b*). The bath is filled with liquid N_2_ up to the cover plate to keep the tripod with the vitrified samples (left), the sample-transfer station (middle) and the puck with cryovials cold. Large forceps, a rod and a manipulation tool are stored in the upper right corner. The extensions for elongation of the sample unit to form the sample holder are stored at the top middle. To separate a sample unit from the cartridge it is slid into the sample-transfer station. This is performed with the forceps, with the cartridge facing downwards. Using the rod, the cartridge can be held in place (small bore). With the manipulation tool, an extension is taken (magnetic) and placed onto the sample unit. The sample holder is now complete and is transferred to the cryovial in the puck. The cartridge is removed from the transfer station with the manipulation tool and a new transfer cycle can be started.

The high-pressure cooler was based on the Leica EM ICE system (Vienna, Austria, see Fig. 3 ▸ *a*), which was modified to fit the needs of protein crystallography. Sample units were inserted into a cylindrical cartridge in the loading station. When a cooling cycle was started, the sample unit together with the cartridge was transferred to a high-pressure chamber, where the sample was cooled with pressurized liquid nitrogen. At a sensor placed in front of the high-pressure chamber, the maximum pressure of 220 MPa was attained after 18 ms and the minimum temperature of 110.5 K was achieved after 420 ms. The pressure in the high-pressure chamber was released after 315 ms (see Supplementary Fig. S2). After a cooling cycle had been initiated on the modified Leica EM ICE system, it took 4 s to complete the following procedure: the cartridge with the sample unit was picked up, moved to the high-pressure chamber, high-pressure cooled and dropped into the sample-storage dewar, where it was kept in liquid N_2_. The next cooling cycle could be started after a recovery time of about 60 s, which was necessary to build up the working pressure of the machine.

The major modifications of the newly built prototype with respect to the EM ICE were a customized loading mechanism, modified cartridges and the described sample holders. This equipment currently cannot be used for standard EM sample preparation. A combination of both instrument types is under consideration.

### 2.4. Sample transfer   

A semi-dry liquid N_2_ bath was designed to detach the sample unit from the cartridge and simultaneously attach the extensions to it. The complete sample holder was then transferred into a cryovial. The semi-dry liquid N_2_ bath consists of a container and a cover plate that provide space for all of the materials and tools that are needed (see Fig. 3 ▸ *b*). The bath was partly filled with liquid N_2_; the inlet plate was sitting directly above the liquid N_2_ level. Thus, gaseous nitrogen was formed in the top half of the container, resulting in a two-phase system. In this way, the preparation of samples took place in a cold and dry environment, while the samples were stored in a puck that was sitting inside liquid N_2_. After all cooled samples had been transferred they could be stored as usual.

### 2.5. Cooling tests of crystallization-screen ingredients   

**Figure 4:**
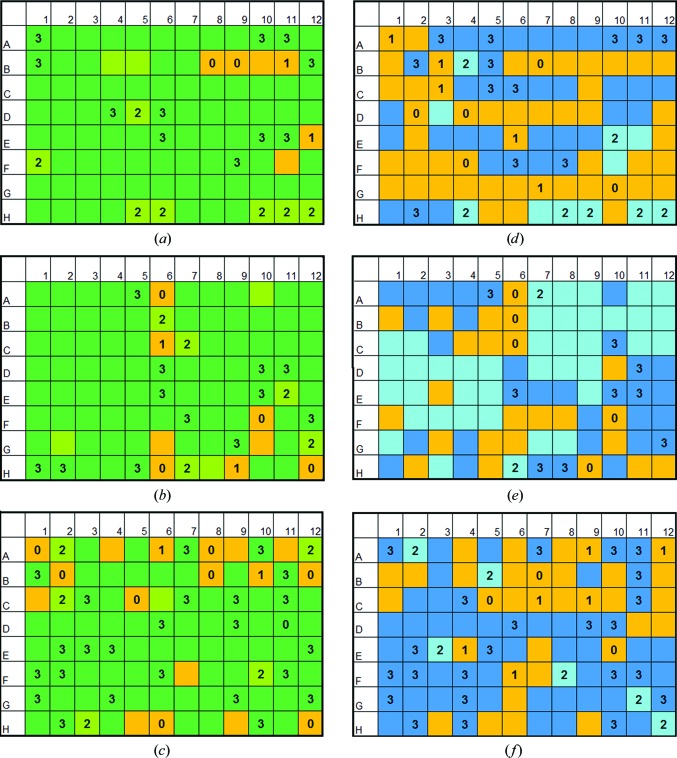
Survey of the cooling tests. Illustrated are the three crystallization screens tested: JBScreen Classic HTS I (Jena Bioscience; top), The JCSG Core Suite I (Qiagen; middle) and MemGold ECO (Molecular Dimensions; bottom). On the left are the HPC results: (*a*), (*b*) and (*c*). On the right are the results for gas-stream cooling: (*d*), (*e*) and (*f*). Boxes corresponding to the respective solutions were filled with the count of samples that formed amorphous ice during the cooling tests. Boxes without numbers were extrapolated from the screening results. The boxes were marked in orange if one or more trials out of three replicates were not cooled successfully. Where two out of three samples showed amorphous ice, boxes were coloured light green (HPC) and light blue (gas stream). For all solutions which showed amorphous ice in all samples the boxes were coloured green (HPC) and blue (gas stream).

A concentration range of different precipitants was tested. These covered PEGs, polyols and salts. For this purpose, three commercially available screens were chosen that were used most frequently at the crystallization facility of the Max Planck Institute of Biophysics (Thielmann *et al.*, 2012 ▸). The complex chemical mixtures originated from the screens The JCSG Core Suite I (Qiagen, Hilden, Germany), MemGold ECO (Molecular Dimensions, Suffolk, England) and JBScreen Classic HTS I (Jena Bioscience, Jena, Germany). The crystallization-screen ingredients per well can be downloaded from the websites of the manufacturers (https://www.qiagen.com, https://www.jenabioscience.com and https://www.moleculardimensions.com), our website (https://registration.cc.biophys.mpg.de/pickscreens/; Hedderich *et al.*, 2011 ▸) or others (http://c6.csiro.au; Newman *et al.*, 2010 ▸). All 288 conditions were downloaded from PICKScreens and sorted based on their precipitant. The main component of the solution was chosen to be the precipitant. In cases where the solutions contained 2-propanol and PEG 4000, the mixtures were evaluated. If three or fewer concentrations per precipitant were present in the screens, all of them were chosen to describe the minimum concentration needed for amorphous ice formation in cooling and HPC. If more solutions with varying precipitant concentrations existed in the screens, low concentrations were chosen to describe the minimum concentration per precipitant necessary to yield amorphous ice formation during cooling. All solutions were directly taken from the deep-well blocks of the corresponding screens. Each solution was cooled individually three times. For HPC samples the screen solution was absorbed into the sample unit (about 100 nl), closed with a saturated PEG 100 000 solution, cooled as depicted in §[Sec sec2.3]2.3 and tested as described in §[Sec sec2.6]2.6. A total of 255 samples were cooled by HPC. An overview of the results can be found in Figs. 4 ▸(*a*), 4 ▸(*b*) and 4 ▸(*c*). Amorphous ice was usually formed by HPC, as shown in Supplementary Fig. S3(*c*): the maximum of the diffuse scattering shifted to a lower *d*-spacing compared with the inner cubic ice ring. This indicated the formation of HDA ice, as shown by Kim *et al.* (2008 ▸).

If no samples or one sample out of three could be cooled to form amorphous ice, the solutions were counted as not cooled successfully (orange in Fig. 4 ▸; if a solution in a well position was chosen for measurement, the counts for successful cooling are depicted in each well position for the three different screens). A transition zone for cooling was observed if only two out of three samples formed amorphous ice (light green in Fig. 4 ▸). If a solution could be cooled in three out of three samples the solution was counted as cooled successfully (green in Fig. 4 ▸). All solutions with the same or an increased precipitant concentration as a successfully cooled solution were extrapolated as suitable for HPC (the well position for the solution was marked in green without numbering). Solutions which had precipitant concentrations in between two not successfully cooled solutions or had the same or a lower precipitant concentration as not successfully cooled solutions were extrapolated as not cooled successfully. Solutions in between two probed solutions or solutions with the same precipitant concentrations where two out of three samples formed amorphous ice were extrapolated as well positions in the transition zone.

Samples for conventional cooling were directly cooled in 150 µm MicroMounts (MiTeGen, Ithaca, New York, USA) in the 100 K Cobra cryostream (Oxford Cryosystems, Oxford, England). The gas stream was blocked until the sample was mounted and suddenly released. The time required to transport a drop of solution in a MicroMount from a well to the goniometer and to release the gas stream was less than 3 s. The liquid volume within a 150 µm sample aperture of a MicroMount is small (about 130 pl) compared with a standard loop as excess liquid is drained into a cavity. For the cooling tests with the gas stream the following references were used to guide the cooling test: Garman & Mitchell (1996 ▸), McFerrin & Snell (2002 ▸) and Rubinson *et al.* (2000 ▸). In general, the tests were started with the higher precipitant concentrations in the solutions for gas-stream cooling. A total of 249 samples were examined. Evaluation and extrapolation of the results was performed as described for HPC. The results are visualized in Figs. 4 ▸(*d*), 4 ▸(*e*) and 4 ▸(*f*) (orange for not cooled successfully, light blue for the transition zone and blue for successfully cooled solutions; the count in the well positions depicts the sample number for amorphous ice formation).

### 2.6. Data collection and evaluation of cooling tests   

Diffraction images of high-pressure cooled lysozyme and concanavalin A crystals were collected on the PXII beamline of the Swiss Light Source at the Paul Scherrer Institut, Villigen, Switzerland.

Diffraction tests of the crystallization-screen ingredients were carried out with an in-house Rigaku FR-E+ SuperBright generator combined with a Saturn 944+ CCD detector (Rigaku Europe, Sevenoaks, England). All diffraction tests were analyzed with respect to the formation of an amorphous state. Examples for diffraction tests with amorphous ice and with crystalline ice rings can be found in Supplementary Fig. S2.

### 2.7. Data processing and analysis   

The diffraction patterns were indexed, integrated and scaled using *XDS* (Kabsch, 2010 ▸). Molecular replacement was performed using *Phaser-MR* within the *PHENIX* package. Manual rebuilding in *Coot* and refinement in *PHENIX* were used alternatingly (Adams *et al.*, 2010 ▸; Emsley & Cowtan, 2004 ▸).

## 3. Results   

### 4. Technical realization   

The most important requirement for crystal handling is a safe and reliable system. Initially, we tried to cool crystals on loops, but the unpredictable position of the crystal in the cylindrical cartridge or on the steel pin of the cryoloop forced us to change the surrounding of the crystal to a capillary. This change increased the reliability of the method drastically. From these findings, a sample unit was developed that holds the capillary during cooling (see Fig. 2 ▸ *a* and Supplementary Fig. S1 for dimensions). In order to create an easy and straightforward loading mechanism for HPC, it was absolutely necessary to change the setup from half-cylinders (Leica EM ICE) to cylinders (see Fig. 2 ▸ *d*). This alteration enabled loading that could be effectively guided by tools with no need for supervision by eye. The most important invention was a setup for the cylindrical cartridge that allowed proper cooling of the crystals within the mother liquor in the capillary, and kept the vitrified material cool during the drop from the cooling chamber into the sample dewar. It was an iterative process to optimize the geometry of the cylindrical cartridge by influx and efflux bore diameter, the positioning of the sample, the material thickness and the cylinder material itself. In addition, the cartridge needed to be adapted as the sample unit progressed to the final state. It can then be elongated by an extension to fit the SPINE standard for automation at European synchrotrons. The assembly of the sample unit and the cylindrical cartridge is held together by two magnets to keep the sample in the capillary in a safe position during the whole cooling and manipulation process.

#### 4.1. Workflow of HPC   

A method to cryoprotect crystals with high pressure should be as simple as cooling with regard to manual manipulation. Therefore, special care was taken to develop a simple process that can be divided into three steps.

(i) *Harvesting of crystals*. Crystals are harvested in a polyimide capillary of the sample unit by using capillary forces (Fig. 2 ▸). The polyimide capillary is sealed with PEG 100 000 solution^2^.
(ii) *High-pressure cooling*. Insertion of the sample unit into the cartridge. The cartridge is already preloaded in the loading station of the modified Leica EM ICE (Fig. 3 ▸ *a*). Finally, the user initiates the loading of the cartridge into the high-pressure chamber.
(iii) *Preparations for sample storage*. The cartridge and sample unit are taken from the sample dewar of the modified Leica EM ICE to the semi-dry liquid N_2_ bath (Fig. 3 ▸ *b*). Here, the cartridge is separated from the sample unit. In the same step the sample unit is also elongated by an extension to form the sample holder. The sample holder is kept in a cryovial in liquid N_2_ for storage.

#### 4.2. Useful cooling range   

To evaluate how useful HPC is compared with the conventional cooling approach, a rather extensive screening of crystallization solutions was performed. The three most frequently used screens at our large crystallization facility for membrane proteins were tested with 83 (gas-stream cooling) and 85 (HPC) out of 288 crystallization solutions. The screens were The JCSG Core Suite I (Qiagen), MemGold ECO (Molecular Dimensions) and JBScreen Classic HTS I (Jena Bioscience). The evaluation criteria were as follows. If all three individually cooled samples showed no ice rings, the condition was deemed to be suitable for HPC or gas-stream cooling (see Fig. 4 ▸). A transition zone for cooling was attained when only two out of three samples were cooled properly. From the 85 (HPC) investigated solutions the results for the remaining 203 (HPC) solutions were extrapolated as described in §[Sec sec2.5]2.5. In Fig. 4 ▸ all well positions which were investigated show the count of successfully cooled samples. Extrapolated well positions are marked by the corresponding colour only. When cooling is applied in the gas stream in 150 µm MicroMounts, 47.3% of the crystallization solutions should form amorphous ice in all three samples. Additionally, 19.8% of the solutions should show amorphous ice in two out of three samples. In comparison, HPC should be able to cool the crystallization solutions in more than 81.2% of the tests successfully. Moreover, another 8.0% of the solutions should be cooled in two out of three samples; this would result in a total of 89.2% of the solutions. If both methods, HPC and gas-stream cooling, were merged for all conditions giving at least two out of three successfully cooled samples, a total of 90.2% of the tested conditions could be used.

**Figure 5:**
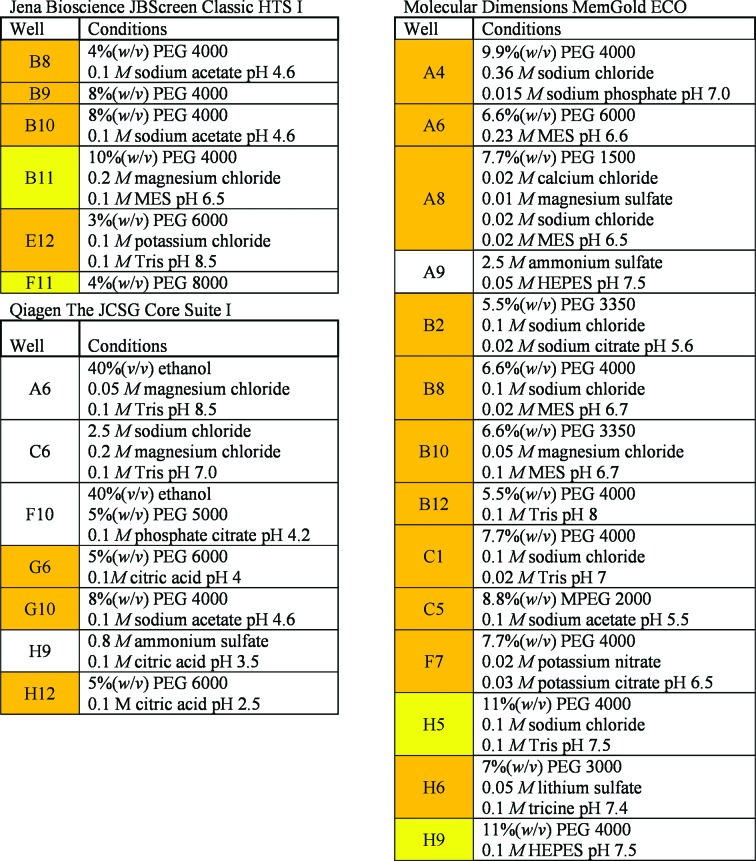
Overview of the solutions that were not well suited for HPC or cooling from Fig. 4 ▸. Screening solutions from the respective screens that failed to be cooled to amorphous ice are listed. Wells marked in yellow can be used for HPC if the PEG concentration is raised by 1–2%(*w*/*v*). Conditions marked in orange can be cooled by HPC if the PEG concentration is further increased by up to 8%(*w*/*v*). The remaining five of the 288 conditions need conventional treatment with additional cryoprotectants.

The precipitant solutions that could be vitrified only by gas-stream cooling were all from MemGold ECO (bottom in Fig. 4 ▸; A1, A11 and H12). All of these precipitant solutions contained ammonium sulfate or sodium chloride. All other solutions that could not be vitrified by either method are listed in Fig. 5 ▸. By inspection of the successful and unsuccessful cooling trials for HPC it was possible to deduce predictions of the cooling results. If small-sized PEGs were used with an average molecular weight of 200–600 Da, all solutions formed amorphous ice. The lowest concentration tested was 15%(*w*/*v*). For medium-sized PEGs of between 1000 and 6000 Da, a concentration of 11–12%(*w*/*v*) was needed to form amorphous ice in the samples. For PEG 8000 a concentration of only >5%(*w*/*v*) was needed for amorphous ice formation in the samples. Larger PEGs (10 000 and 20 000) formed amorphous ice at the lowest concentrations of 8–10%(*w*/*v*) in the solutions. The cooling was mostly dependent on the chain length of the PEG; it did not vary when monomethyl ethers were used. Jeffamines, pentaerythritol propoxylate, triethylene glycol and 2-methyl-2,4-pentanediol were present in these screens only at high concentrations that exceeded the limit necessary for forming amorphous ice. It seems reasonable that a similar dependence of required concentrations on chain lengths might apply. For salts we were not able to deduce a general criterion for successful cooling. Lithium sulfate (1.0 *M*) and sodium citrate (1.1–1.6 *M*) were cooled successfully by HPC. A mixture of potassium and sodium phosphate at 0.8 *M* was still cooled in two out of three samples. Samples containing ammonium sulfate (0.8–2.5 *M*) and sodium chloride (2.5–3.0 *M*) were cooled at most in only one out of three cases with HPC. Interestingly, these were partly cooled by gas-stream cooling.

The screen solutions that were not eligible for cooling (shown in Fig. 5 ▸) can be classified into two groups. One group can be easily rescued by applying the above criteria for PEGs. This observation means that for all conditions coloured yellow a concentration increase of 1–2%(*w*/*v*) is sufficient to properly cool these solutions. Solutions coloured orange can be cooled if the concentration of the PEG is further increased by up to 8%(*w*/*v*). The remaining noncoloured conditions form the second group, which is not appropriate for both HPC and gas-stream cooling. These comprise only five out of 288 conditions, and contain ethanol, sodium chloride and ammonium sulfate. The rather high concentrations of pure alcohols from 20 to 40%(*v*/*v*) cannot be cooled by HPC and should be conventionally treated by the addition of cryoprotectants. Mixtures of alcohols with PEGs of appropriate concentration are suitable for HPC [JBScreen HTS I, well D4: 10%(*w*/*v*) PEG 4000, 20%(*v*/*v*) 2-propanol]. A solution containing glycerol was present in the screens at a minimal concentration of 22%(*v*/*v*) as precipitant and could be cooled without difficulty.

#### 4.3. Comparison of model proteins   

**Figure 6:**
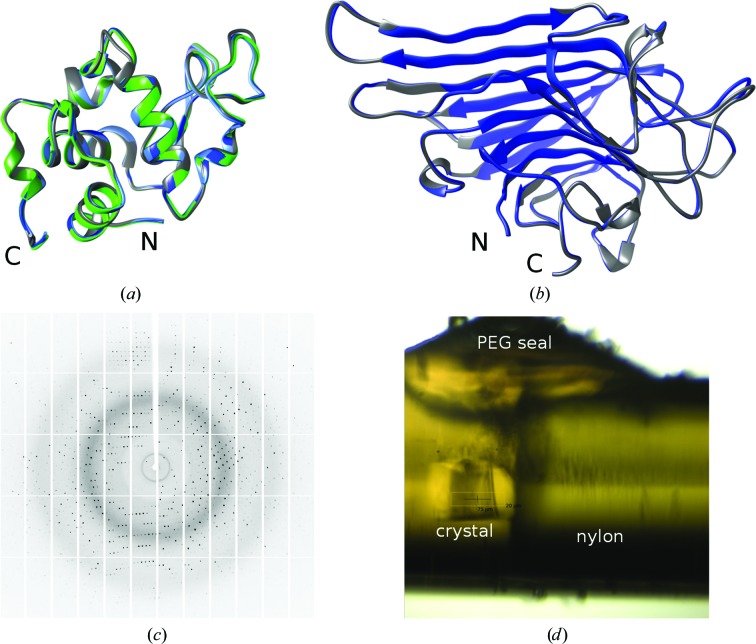
From the crystals to the structures. The crystal structures compared in Table 1 ▸ are superimposed for lysozyme (*a*) and concanavalin A (*b*). The colour code in (*a*) is as follows: PDB entry 4wld, light green; 4wlt, dark green; 4a7d, light blue; 5lyt, dark grey; 5o6q (this study), blue. In (*b*) PDB entry 1jbc is coloured dark grey and 5o6n (this study) blue. For both proteins the crystal structures superimpose very well for the main-chain atoms. (*c*) The diffraction pattern of concanavalin A. (*d*) The polyimide capillary with HPC-treated crystal is shown as it was visible at the PXII beamline microscope. The nylon thread can be seen on the right. It restricts the volume of the capillary. On top of the polyimide capillary is the PEG 100 000 seal covering the cavity.

Lysozyme and concanavalin A were crystallized in conditions with 0.8 *M* NaCl and 6%(*w*/*v*) PEG 8000 as precipitants. The crystals that were formed had a size of about 100 µm for each crystal dimension. Diffraction data were collected to 1.45 and 1.35 Å resolution, respectively. Detailed tables of data statistics and model refinement are available in Supplementary Table S1 for lysozyme (PDB entry 5o6q) and in Supplementary Table S2 for concanavalin A (PDB entry 5o6n). The statistics for this study compared with deposited structures of lysozyme and concanavalin A obtained at different temperatures and applied pressures are compiled in Table 1 ▸. For lysozyme a variety of structures are available. Data sets were obtained at ambient pressure and room temperature (PDB entry 4wld), pressurized and room temperature (190 MPa; PDB entry 4wlt), cooled to 100 K and ambient pressure (PDB entry 5lyt), and pressurized and cooled to 100 K (210 MPa; PDB entry 4a7d and this study). All compared structures were determined using crystals in space group *P*4_3_2_1_2. By comparing the unit-cell dimensions a clear shrinkage of the unit-cell volume of 4.3% can be calculated from 4wld to 5lyt. This reduction in the unit-cell volume is only caused by cooling to 100 K. The reduction of the unit-cell volume caused by pressurization to 190 MPa is less prominent at only 2% (4wld to 4wlt). If pressurization and cooling are combined in this study, a shrinkage of 4.8% of the unit cell can be calculated (4wld to 5o6q). This value is similar to the shrinkage induced by cooling of the crystal with additional cryoprotectants. Differences in the unit-cell volume could also be slightly affected by dehydration of the crystals. All structures were determined at similar resolutions, are similarly well refined and have a low r.m.s.d. of less than 0.41 Å for the main-chain atoms compared with PDB entry 5o6q (this study; see Fig. 6 ▸ *a*).

The structure comparison for concanavalin A is based on data collected at 120 K and ambient pressure [PDB entries 1jbc and 5o6n (this study)]. Both crystals belonged to space group *I*222. The unit-cell volume is almost identical for both crystals. The resolution for both structures is high at 1.15 and 1.35 Å, respectively. Both structures are similarly well refined and show a main-chain r.m.s.d. of only 0.23 Å (see Fig. 6 ▸ *b*).

## 5. Discussion   

We have performed numerous measurements with gas-stream cooling and they performed better than our previous experiences with conventional plunge-cooling. In cases where a crystal cannot just be cryoprotected by plunge-cooling in its mother liquor, it might be advisable to also work on the cooling technique. One easy way might be to use a nitrogen gas stream. Another approach could be to remove the cold gas layer above the liquid nitrogen, which has been termed hyperquenching by Warkentin *et al.* (2006 ▸).

Since 1973 it has been known that cooling at high pressure could be an alternative to the cryoprotection of protein crystals directly from their mother liquor. Structures of crystals cooled at high pressure have been published by different groups to show its applicability to various biological systems. However, the different procedures included many long steps or very complex handling. The methods presented by Kim *et al.* (2006 ▸) and van der Linden *et al.* (2014 ▸) used helium gas and needed a time of 5–25 min for pressurization. Burkhardt *et al.* (2012 ▸) and Kurz *et al.* (2012 ▸) used a high-pressure cooler developed in the field of EM. Before crystals in quartz capillaries could be cooled in the setup, they had to be placed in EM sample holders. The high-pressure chamber had to be filled with ethanol. Extensive manual manipulation was required before and after cooling. The current procedure combines the strengths of both approaches. A sample holder suitable for crystals and X-ray analysis has been developed and can be used in a rapid cooling process which only requires pressurized liquid nitrogen. The workflow of the HPC procedure is rapid and kept rather simple, avoiding complex manipulation steps.

The HPC technique offers a direct approach to the uniform cooling of solutions in 250 µm polyimide capillaries. In contrast, in a study by Warkentin *et al.* (2008 ▸) capillaries of a similar diameter were plunge-cooled according to the hyperquenching method and needed a concentration of 30–35%(*w*/*v*) glycerol to appear transparent when cooled. Using HPC, greater than 89% of the examined crystallization solutions form amorphous ice and appear visually clear (this also includes the narrow transition zone of 8.0%). The percentage of solutions cooled to an amorphous state can be further increased if the PEG concentrations are minimally raised by up to 8%(*w*/*v*) in the crystallization solution and cooled by HPC. More than 97% of the conditions could then be cooled. The initial target for HPC was the model protein lysozyme. Interestingly, we never had any problems in cooling lysozyme crystals in their mother liquor 0.8 *M* sodium chloride, 0.1 *M* sodium acetate pH 4.6. From the results presented above this condition should be rather difficult for HPC. However, the mother liquor also contained the remaining lysozyme and this might be the important difference. The protein left in the crystallization solution may facilitate the cooling process and HPC could be applicable even to a lower range of precipitant concentrations than described here. The HPC technique introduced represents enormous savings in time, cost and resources when the cryoprotectants are not known or when the crystals are difficult to handle in the presence of additional cryoprotectants.

The treatment of the crystals in HPC was rather mild. The crystals were harvested in a single procedure and were safe in the capillary. The position of the crystal could be inspected using a microscope. There was sufficient time to seal the tip and the cavity at the side of the capillary with a solution of high-molecular-weight PEG. This procedure prevented optical disturbances from whirls and bubbles in the capillary that were created if the capillary was not sealed with PEG. The mother liquor of the crystal was kept inside the capillary. The crystal was not stressed by chemical perturbations.

The handling and harvesting in capillaries was different from previous methods and needed to be practiced. This statement was especially true if the crystals were harvested from 96-well plates. We are currently working on a procedure to facilitate the harvesting process. The loading of the high-pressure cooler and the handling in the semi-dry liquid N_2_ bath were guided by tools and were very accurate. The cooling procedure worked rapidly and reliably. We were able to high-pressure cool nine crystal samples in 10 min. An additional time of about 30 s per sample unit was needed to remove the cartridge and add the extension. About 90% of the high-pressure cooled crystals could be directly visualized on the microscope of the PXII beamline at the Swiss Light Source. More crystals were found by performing a grid screen with the X-ray beam. A very important improvement was a piece of nylon in front of the copper and inside the polyimide capillary. This addition limited the end of the capillary to which the crystals could be absorbed and prevented the possibility of scattering from the copper.

For the two model proteins lysozyme and concanavalin A the complete HPC procedure showed very similar results compared with known crystal structures. For lysozyme all crystal structures from the different physical settings had a corresponding main-chain r.m.s.d. of less than 0.41 Å.

An interesting finding was that the majority of crystallization conditions that were not cooled to amorphous ice showed cubic ice rings and were optically clear. The X-ray diffraction analysis was absolutely necessary to probe this. Moreover, we were not able to cool pure alcohol–water mixtures to amorphous ice. From the analysis of a powder diffraction pattern of 20%(*v*/*v*) ethanol cooled by HPC, we suspect the formation of ice II and ice III in the capillary. Apparently, the crystallization kinetics play a role depending on the additives used in the screen solutions. As stated above, we are working on a variation of the sample unit to simplify the harvesting process. We would also like to dispense the liquid from the capillary once the crystal has been taken up. This should improve the signal-to-noise ratio for all X-ray measurements. Another important fact is that 96-well plates often contain drops with 100–200 nl volume. If one crystal-harvesting attempt has already used all of the mother liquor (100 nl for one capillary), then the remaining crystals cannot be withdrawn. This is highly problematic if membrane proteins are handled, where the exact concentration of the detergent in the mother liquor is not known and cannot be replaced. The first trials with capillaries and crystals without mother liquor have been successful. A tool to easily operate the new mechanism is currently being built.

## 6. Conclusion   

HPC has been developed as a very rapid and reliable technique to directly cryopreserve crystals in their mother liquor. A wide chemical space of crystallization conditions can be homogeneously cooled. Structure determinations of high-pressure cooled crystals of model proteins lead to very similar structures compared with crystals analyzed using commonly employed methods. Extensive trials are ongoing to investigate whether the procedure can also be advantageous for challenging and sensitive crystals, for example for membrane proteins.

## Supplementary Material

PDB reference: concanavalin A, 5o6n

PDB reference: lysozyme, 5o6q

Supplementary Figures and Tables.. DOI: 10.1107/S2059798317016357/nj5271sup1.pdf

## Figures and Tables

**Table 1:** Comparison of crystal structures The following abbreviations are used in the table: *T*, temperature; *p*, pressure; r.m.s.d., root-mean-square difference of the main-chain atoms calculated by the least-squares superpose tool in *Coot* with respect to PDB entry 5o6q or 5o6n; RT, room temperature.

Structure	Space group	*a*, *b*, *c* (Å)	*T* (K)	*p* (MPa)	*d*_min_ (Å)	*R*/*R*_free_	R.m.s.d. (Å)	Reference
Lysozyme								
4wld	*P*4_3_2_1_2	79.20, 79.20, 37.90	RT	0.1	1.54	0.148/0.179	0.24	Yamada *et al.* (2015 ▸)
4wlt	*P*4_3_2_1_2	78.22, 78.22, 38.04	RT	190	1.6	0.153/0.197	0.19	Yamada *et al.* (2015 ▸)
5lyt	*P*4_3_2_1_2	78.42, 78.42, 36.98	100	0.1	1.9	0.176	0.41	Young *et al.* (1993 ▸)
4a7d	*P*4_3_2_1_2	77.22, 77.22, 37.01	100	210	1.5	0.175/0.203	0.18	Burkhardt *et al.* (2012 ▸)
5o6q	*P*4_3_2_1_2	77.95, 77.95, 37.27	100	220	1.45	0.172/0.197		Current work
Concanavalin A								
1jbc	*I*222	61.95, 86.05, 89.08	120	0.1	1.15	0.142/0.167	0.23	Parkin *et al.* (1996 ▸)
5o6n	*I*222	61.26, 86.23, 89.02	100	220	1.35	0.133/0.164		Current work

## Footnotes

1 This procedure differs from harvesting with loops as the harvesting attempt is finished once the capillary has filled completely.

2 The PEG seal is used to optimize the visibility in the capillary, as whirls and bubbles can mask crystals.
