# Supplementary material for: A standardized technique for high-pressure cooling of protein crystals
Source: Acta Crystallogr D Struct Biol. 2017 Nov 22;73(Pt 12):997–1006. doi: 10.1107/S2059798317016357 (PMC6116161; doi:10.1107/S2059798317016357)
Supplement: Supplementary file 1 [file d-73-00997-sup1.pdf]

STRUCTURAL BIOLOGY

Volume 73 (2017)

Supporting information for article:

A standardized technique for high-pressure cooling of protein crystals

David Quirnheim Pais, Barbara Rathmann, Juergen Koepke, Cveta Tomova, Paul Wurzinger and Yvonne Thielmann

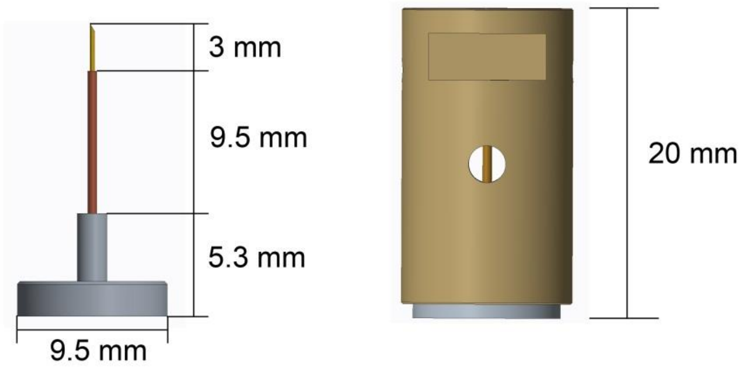

Figure S1 Schematic with dimensions for the sample unit (left) and the sample unit with the cartridge (right). After the extension (5.2 mm height) is placed onto the sample unit the sample holder is complete. It has a length from bottom of extension to the top of the capillary of 23 mm.

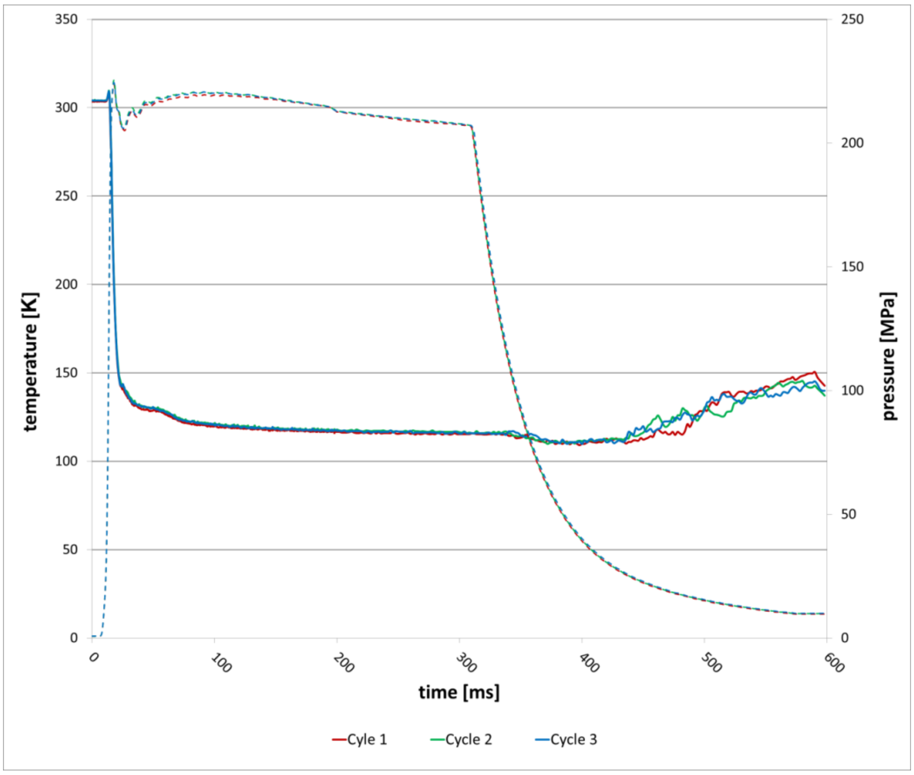

Figure S2 Temperature and pressure curves of three consecutive high pressure cooling cycles. The sensors for pressure and temperature are placed closely in front of the high pressure chamber. The pressure curve is indicated by a dashed line and the temperature curve by a solid line. In the modified Leica EM ICE the pressure rises from ambient pressure to more than 220 MPa in 18 ms and drops after 315 ms back to 10 MPa. The temperature drops from 304 K to the minimum temperature of 110 K after 420 ms.

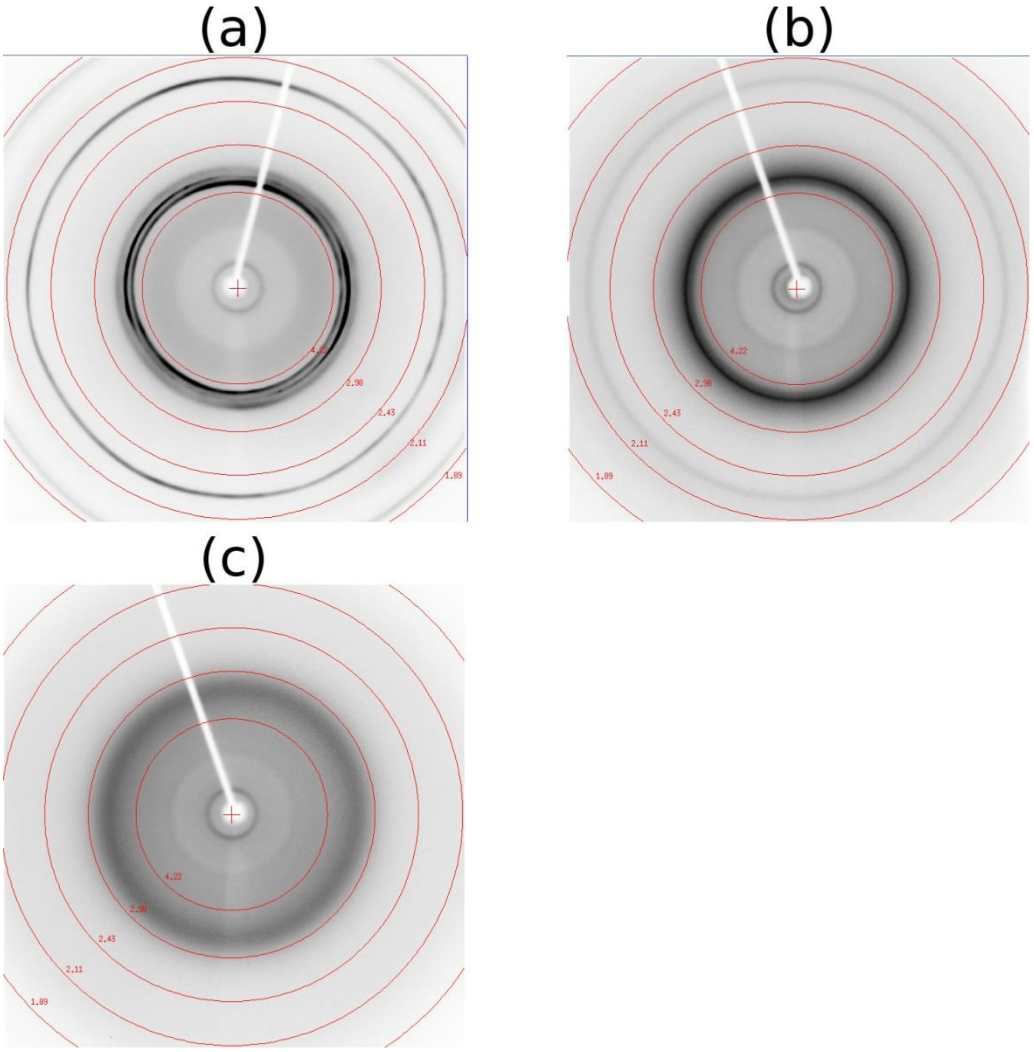

Figure S3 Results for the HPC tests. (a) A sample showing hexagonal and cubic ice rings. (b) A sample showing only strong cubic ice rings. (c) A well cooled sample with amorphous ice and background scattering of the polyimide capillary.

Table S1 Data collection and refinement statistics of 5o6q.

	**5o6q**
Wavelength (Å)	1.000
Resolution range (Å)	38.97 - 1.449 (1.501 - 1.449)
Space group	P 43 21 2
α, *β*, *γ* (Å)	77.95, 77.95, 37.27
Mosaicity (°)	0.165
Total reflections	524512 (49524)
Unique reflections	20965 (2054)
Multiplicity	25.0 (24.1)
Completeness (%)	98.21 (92.27)
Mean I/sigma(I)	36.29 (1.95)
Wilson B-factor ($Å^2$)	22.88
R-pim	0.0098 (0.381)
CC1/2	1.000 (0.744)
CC*	1.000 (0.924)
Reflections used in refinement	20591 (1899)
Reflections used for R-free	1970 (185)
R-work	0.1724 (0.2916)
R-free	0.1969 (0.3311)
CC(work)	0.969 (0.815)
CC(free)	0.965 (0.749)

Number of non-hydrogen atoms	1190
macromolecules	1046
ligands	9
solvent	135
Protein residues	129
RMS bonds (Å)	0.005
RMS angles (°)	0.76
Ramachandran favored (%)	98.43
Ramachandran allowed (%)	1.57
Ramachandran outliers (%)	0.00
Rotamer outliers (%)	0.88
Clashscore	6.24
Average B-factor ($Å^2$)	32.43
macromolecules	31.43
ligands	35.78
solvent	39.95
Number of TLS groups	9

Statistics for the highest-resolution shell are shown in parentheses.

Table S2 Data collection and refinement statistics of 5o6n.

	**5o6n**
Wavelength (Å)	1.000
Resolution range (Å)	33.23 - 1.348 (1.396 - 1.348)
Space group	I 2 2 2
α, *β*, *γ* (Å)	61.26, 86.23, 89.02
Mosaicity (°)	0.173
Total reflections	302898 (14025)
Unique reflections	51600 (4565)
Multiplicity	5.9 (3.1)
Completeness (%)	97.26 (81.49)
Mean I/sigma(I)	20.83 (2.42)
Wilson B-factor ($Å^2$)	12.49
R-pim	0.0194 (0.2418)
CC1/2	0.999 (0.854)
CC*	1.000 (0.960)
Reflections used in refinement	50806 (4196)
Reflections used for R-free	1968 (158)
R-work	0.1327 (0.2213)
R-free	0.1639 (0.2306)
CC(work)	0.980 (0.924)
CC(free)	0.967 (0.842)

Number of non-hydrogen atoms	2366
Number of non-hydrogen atoms	2366
macromolecules	1929
ligands	5
solvent	432
Protein residues	237
RMS bonds (Å)	0.005
RMS angles (°)	0.82
Ramachandran favored (%)	98.72
Ramachandran allowed (%)	1.28
Ramachandran outliers (%)	0.00
Rotamer outliers (%)	1.30
Clashscore	3.84
Average B-factor ($Å^2$)	18.89
macromolecules	15.75
ligands	17.65
solvent	32.90

Statistics for the highest-resolution shell are shown in parentheses.
